# Umbilical acupuncture combined with warm needle acupuncture for the treatment of residual numbness after lumbar disc herniation surgery: a protocol of a randomized controlled trial

**DOI:** 10.3389/fneur.2025.1528411

**Published:** 2025-03-11

**Authors:** Kaihua Song, Jiayue Cheng, Yuming Wang, Yang Shen, Chenxin Jiang, Shouhai Hong

**Affiliations:** ^1^The First Clinical Medical College of Zhejiang Chinese Medical University, Hangzhou, Zhejiang, China; ^2^The First Affiliated Hospital of Zhejiang Chinese Medical University, Hangzhou, Zhejiang, China

**Keywords:** lumbar disc herniation, postoperative residual numbness, randomized controlled trial, protocol, umbilical acupuncture, warm needle acupuncture

## Abstract

**Background:**

Postoperative residual numbness following lumbar disc herniation (LDH) surgery is a relatively common symptom. Existing drugs and physical therapies are not very effective. Acupuncture is effective for the treatment of LDH, but there are limited reports on its use for postoperative numbness. Based on clinical observations and literature review, we hypothesize that combining umbilical acupuncture (UA) with warm needle acupuncture(WA) may yield superior efficacy compared to electroacupuncture(EA) alone, enhancing immune function, promoting nerve recovery, and improving microcirculation through synergistic effects, thereby filling the gap in this treatment field.

**Objective:**

This proposed trial aimed to evaluate the effectiveness and safety of UA (umbilical acupuncture) combined with WA (warm needle acupuncture) in treating residual numbness after LDH surgery. To verify the hypothesis that the combined method is superior to traditional electroacupuncture.

**Methods:**

This proposed study is a single-center, single-blind, prospective, randomized controlled trial (RCT) involving patients with LDH who were hospitalized and underwent percutaneous endoscopic lumbar discectomy (PELD) at our hospital. Patients meeting the inclusion criteria will be randomly assigned to either the treatment group (umbilical acupuncture combined with warm needle acupuncture) or the control group (electroacupuncture). The participants will be assessed on the first day after surgery, and acupuncture treatment will begin on the second day and continue for three consecutive days, with each session lasting 30 minutes. After that, the treatment mixture was changed three times a week for four weeks. All patients received standard Western medical drug treatment. After the treatment is concluded, a six-month follow-up will be conducted. The primary efficacy indicator will be the visual analog scale (VAS) score for numbness. The secondary efficacy indicators will include the 10 g monofilament test, 40 g pressure acupuncture sensation examination, Japanese Orthopedic Association (JOA) score, lower limb electromyography (H-reflex differences, F-wave conduction velocity, and latency), VAS score, traditional Chinese medicine symptom scoring, and Short Form 36-Health Survey (SF-36) score. Any adverse events occurring during the trial will be recorded. The data will be analyzed according to a predefined statistical analysis plan.

**Discussion:**

This trial combines UA with WA to create a new non-invasive treatment for numbness after LDH surgery, an area where current therapies are inadequate. If proven effective, this combination therapy could offer a safer and more effective alternative to drug treatment, and provide evidence for the integration acupuncture strategies.

**Clinical trial registration:**

http://itmctr.ccebtcm.org.cn/, identifier ITMCTR2024000328.

## 1 Introduction

Patients with lumbar disc herniation (LDH) usually present with symptoms such as pain, numbness, and weakness, which negatively impact their social functioning (1, 2). Studies indicate that the incidence of residual numbness ranges from 10.0 to 40.0% (3, 4), with some studies suggesting the probability can be as high as 75% (5). Differences in surgical techniques, heterogeneity of patient populations, variations in follow-up duration, and discrepancies in study designs can all influence prevalence rates. In recent years, percutaneous endoscopic lumbar discectomy (PELD) has been standardized as a representative minimally invasive spine surgical technique for LDH (6). It offers advantages such as minimal trauma, rapid recovery, and low costs. However, in clinical practice, it is often observed that patients experience improvement in low back pain (LBP) and leg pain (LP) after surgery, they may still have residual leg numbness (RLN) or no relief (7). Persistent numbness and weakness following satisfactory pain relief are commonly seen in patients during the postoperative follow-up (8). Huang’s research found that pain recovery was fastest in the first 6 weeks after surgical decompression, while numbness recovered at a slower pace (8). Another study by Liang et al. involving 334 patients who underwent microscopic surgery for LDH, reported that after 2 years of follow-up, 112 cases of residual leg numbness (LN) were identified, yielding an incidence rate of 33.5% (7). Damage to sensory Aβfibers, which conduct sensations of vibrations and touch, may lead to a general sense of numbness (9, 10). Prolonged symptoms and severe nerve root compression may cause deformation and demyelination of nerve fibers distally, resulting in numbness and weakness (11).

Most studies have focused on the improvement of leg pain (LP) rather than numbness after lumbar decompression surgery (LDS), while the recovery of numbness has been less frequently studied (12). Currently, common treatments include neurotrophic drugs, non-steroidal anti-inflammatory drugs (NSAIDs), and medicinal block therapy; however, their long-term use may lead to undesirable adverse effects and complications (13) and insufficient evidence of effectiveness (14). There is an urgent need to identify more effective treatment options for postoperative residual numbness after LDH. Some studies have shown that acupuncture can effectively treat peripheral neuropathy (PN) caused by various conditions, improve peripheral nerve conduction velocity (NCV), and alleviate symptoms such as numbness, pain, or paresthesia in the limbs (15). Acupuncture activates A-*β*, A-*δ*, and C afferent fibers, with signals ascending through the spinal ventrolateral column to the sensory-motor cortex of the brain (16). It accelerates nerve regeneration (15). Modern medicine surveys found that LDH is treated by electroacupuncture may be through improving local microcirculation, adjusting the abnormal ultra-structure of nerve cells, regulating the immune system, decreasing the level of inflammatory cytokines, influencing the speed of nerve conduction velocity (17). In addition, acupuncture can improve the blood circulation and oxygen supply of the cauda equina, nerve root, and sciatic nerve, promote nerve recovery, and improve symptoms (18–20). Recent research suggests that acupuncture could be a potential treatment option for patients with chronic sciatica from herniated disc (21) and is strongly recommended by international guidelines for lower back pain treatment (22).

In clinical practice, we have found that umbilical acupuncture (UA) effectively mobilizes bodily functions and is characterized by fewer needles, lower pain levels, rapid results, simplicity in operation, safety, reliability, and high patient acceptance, making it a promising new treatment option. Warm needle acupuncture (WA) therapy involves placing moxa on the needle handle to transmit medicinal effects and heat through the needle to the corresponding acupoint. When the needle penetrates the skin, the warmth can transmit deep into the acupoints, effectively alleviating the symptoms of patients’ low back pain and limb numbness (23). Some modern medicine surveys found that LDH can be treated by electroacupuncture by improving local microcirculation, adjusting the abnormal ultrastructure of nerve cells, regulating the immune system, decreasing the inflammatory cytokine levels, and influencing nerve conduction velocity (17).

Therefore, further randomized controlled trials are needed to determine more effective intervention measures for this population. Based on our clinical observations, we found that the intervention group in this trial had a good effect on treating postoperative numbness, but there is a lack of high-quality research. In summary, we have designed a randomized controlled trial to evaluate the efficacy and safety of an innovative method UA (umbilical acupuncture) combined with WA (warm needle acupuncture) compared to electroacupuncture, in alleviating residual numbness after LDH surgery. This aims to provide a non-pharmacological treatment option for patients with residual numbness following LDH surgery and potentially lead to better rehabilitation outcomes for such patients.

## 2 Methods

### 2.1 Design

**Figure 1:**
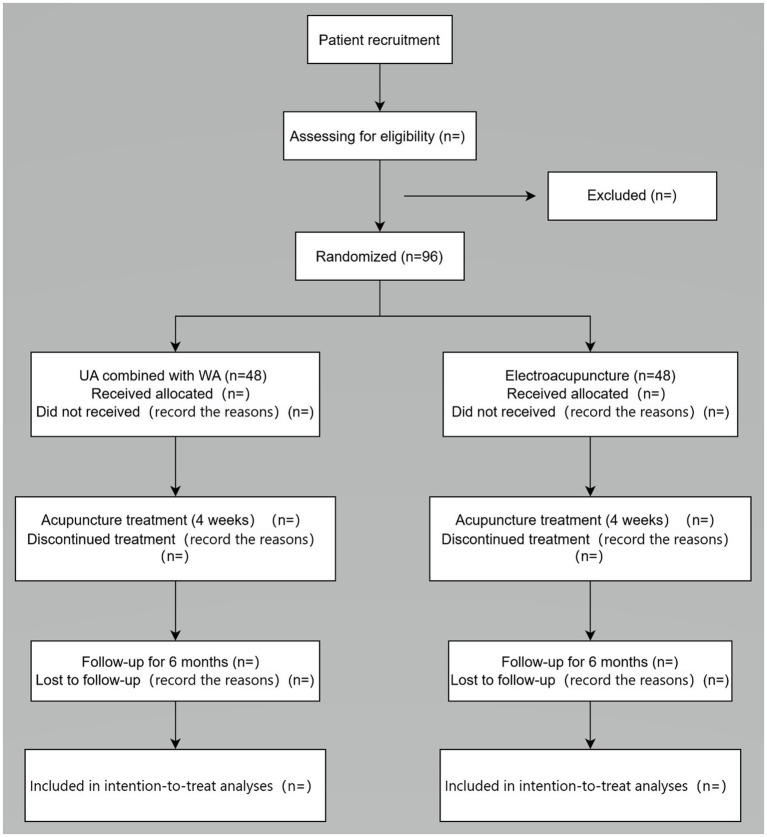
Flow chart of the study procedure.

This study is a prospective, assessor-blinded, randomized controlled trial. A total of 96 participants will be recruited at the Zhejiang Provincial Hospital of Traditional Chinese Medicine. Participants will be randomly assigned to either the experimental group or the control group in a 1: 1 ratio. The trial will consist of a 4-week intervention period followed by a 6-month follow-up period. The flowchart of the research procedure is shown in Figure 1. This study will be designed and reported by the Declaration of Helsinki, the Consolidated Standards of Reporting Trials (CONSORT) (24), and the Standards for Reporting Interventions in Controlled Trials of Acupuncture (STRICTA) guidelines (25). Any potential modifications to the protocol that may affect the conduct of the study will be formally documented as amendments. Before such updates are implemented, they will be decided by the project management team and approved by the ethics committee. The trial has been registered with the Chinese Clinical Trial Registry, number ITMCTR2024000328.

### 2.2 Recruitment

We will recruit inpatients from the Orthopedics and Acupuncture Department (patients transferred to our department for continued treatment due to residual limb numbness after PELD surgery in Orthopedics) as well as outpatients from our hospital. Eligible individuals who agree to participate in the study will sign written informed consent. We will ensure that all participants understand they have the right to withdraw from the study at any stage, and that this will not adversely affect their future treatments. The personal information of participants will be kept confidential and managed by designated professionals on our team.

### 2.3 Participants

#### 2.3.1 Inclusion criteria

(1) Age range from 22 to 60 years old; (2) Patients with lumbar disc herniation who underwent PELD surgery and were assessed to have residual numbness symptoms on the first day after surgery; and (3) Numbness with a VAS score greater than 40 mm (score ≥ 40 mm on a 100 mm Visual Analog Scale [VAS]).

#### 2.3.2 Exclusion criteria

(1) Patients with a history of spinal surgery or severe spinal trauma; (2) Leg neurological symptoms unrelated to LDH, such as cervical spinal cord disease, neurological disorders, or lower extremity vascular diseases; (3) Patients with limb numbness caused by other reasons such as diabetes mellitus; (4) Patients with concurrent bone tuberculosis, tumors, and severe osteoporosis; (5) Patients with concurrent severe diseases affecting the cardiovascular, hematological, digestive, or other systems, or with mental illnesses; and (6) Patients with concurrent autoimmune diseases, allergic diseases, or acute/chronic infectious diseases.

#### 2.3.3 Withdrawal and termination criteria

Withdrawal criteria: (1) Individuals unable to complete the entire treatment and observation process; (2) Individuals who cannot fully understand the various forms and questionnaires provided during the study and require assistance; (3) Individuals concurrently receiving other treatments; (4) Individuals with poor compliance; (5) Individuals with incomplete data affecting efficacy and safety evaluation; (6) Individuals experiencing severe adverse reactions or complications who are unsuitable for continued treatment and are therefore withdrawn from the trial; and (7) Individuals who have not completed more than 2/3 of the treatment course (including those withdrawn from the trial and lost to follow-up).

Termination criteria: Participants experiencing severe adverse events or reactions making it inappropriate to continue in the study; major errors found in the study design or significant deviations from the facts; participants who develop serious complications or deteriorate in a condition requiring emergency measures; participants withdrawing from the clinical study; patients who are uncooperative or noncompliant with treatment, despite repeated explanations from clinicians; during the trial, researchers should retain all original trial records and document in detail the reasons and timing for withdrawal. Those who have completed more than half of the treatment regimen should be included in the efficacy statistics.

### 2.4 Randomization, allocation concealment, and blinding

Eligible patients will be randomly assigned to two groups: the treatment group (receiving warm needle acupuncture combined with Western medicine) and the control group (receiving electro-acupuncture). The randomization will be performed using the CHISS software for block randomization. The size of each block is n. Once the randomization scheme is generated, it will be kept by a third party independent of this study. To ensure allocation concealment, we use sealed opaque envelopes. These envelopes are prepared by an independent member of the research team, and participants draw an envelope randomly upon enrollment in the study to determine their treatment group. Clinical researchers will obtain the specific treatment plan for each patient through designated channels (such as by phone). Assessors will not be informed of the participants’ specific group assignments throughout the follow-up period. All assessment work will be carried out by assessors independent of the treatment process. These assessors will receive rigorous training before the study begins to ensure their standardized understanding and application of the VAS score, thereby ensuring the consistency and accuracy of the assessments. Assessors will be strictly restricted from accessing participants’ treatment group information throughout the follow-up period to maintain assessor blinding. Blind statistical analysis will also be implemented during the data aggregation and analysis phase, with statisticians remaining unaware of the participants’ treatment allocations to ensure the objectivity of the statistical analysis results. In the event of serious adverse events, emergency medical situations, or requests from participants, the sealed envelope containing the blinding information will be opened by an independent third party, and the reasons, time, and personnel involved in the unblinding will be recorded in detail and reported to the relevant committee promptly. Medical decisions will be made based on the unblinding results, and participants will be allowed to withdraw from the trial if necessary.

### 2.5 Sample size

The VAS score is the primary outcome measure of this trial. Based on the preliminary results from the research team’s earlier trials, the estimated VAS score for the treatment group after intervention is 1.6 ± 1 cm. Based on the data from relevant domestic clinical trials (26) and our preliminary exploratory trials, we predict that the VAS score for the control group is 2.41 ± 1.68 cm. The sample size ratio for the two groups is 1:1. Using PASS15 to calculate the sample size, with a two-sided test set at *α* = 0.05 and *β* = 0.10, and a test power of 0.9, at least 41 participants are needed in each group. Considering a 15% dropout rate, a total of 48 participants will need to be recruited for each group, resulting in a total of 96 participants required.

### 2.6 Interventions

Participants in both groups of this study will be assessed on the first-day post-surgery, with treatment beginning on the second day and continuing for three consecutive days, totaling three sessions, with needles retained for 30 min each time. Subsequently, the treatments will be conducted three times a week for 4 weeks. Both treatments will be administered by trained acupuncturists who hold a valid practitioner certificate and have over 5 years of clinical experience, having undergone standardized training before the trial to ensure consistency. After discharge, patients need to undergo acupuncture treatment in the outpatient department; if numbness symptoms disappear, acupuncture treatment should be halted, and follow-up will continue per the experimental protocol.

#### 2.6.1 Intervention group

**Figure 2:**
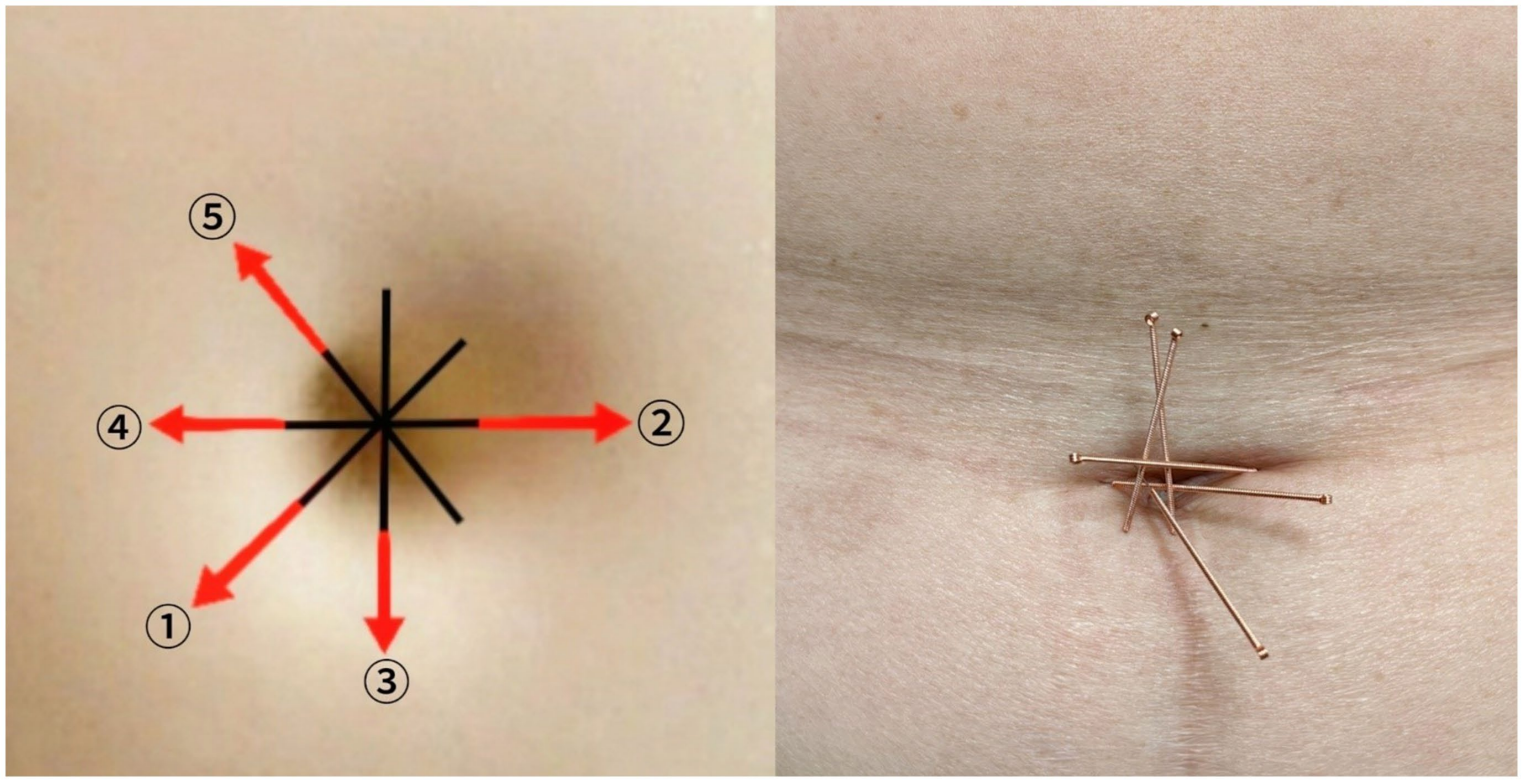
Locations of acupoints.

**Acupoint prescription**: The umbilical acupoint selection is as shown in Figure 2; warm needle acupuncture points are: Qihai (CV 6), Guanyuan (CV 4), the affected side’s Zusanli (ST 36), and Yanglingquan (GB 34) acupoints.

**Needle type**: Disposable sterile acupuncture needles, Jiachen brand.

**Needling technique**: After disinfecting the umbilical area, a 0.25 mm × 25 mm ①②③④⑤ around the umbilicus (see Figure 2), angled radially outward from the center of the umbilicus at approximately 15°, with a depth of insertion around 20 mm, adjusted according to the patient’s body type. Needles are retained for 30 min. Details of selected acupoints are shown in Figure 2.

After routine disinfection of CV 6, CV 4, the affected side’s ST 36, and GB 34, a 0.25 mm × 40 mm acupuncture needle is inserted directly, ensuring “Deqi” sensation is achieved at all acupoints. Once “Deqi” is reached, perform warm needle acupuncture at CV 6, ST 36, and GB 34; moxibustion uses moxa rolls with 1 cm lengths. During moxibustion, cardboard will be used as a shield to prevent burns.

#### 2.6.2 Control group

Based on relevant literature and prior work, the electroacupuncture treatment methodology is as follows:

**Acupoint prescription:** Jiaji (X-B2) acupoints of the lumbar region (affected segment and adjacent segments), Shenshu (BL 23), Dachangshu (BL 25), the affected side’s Huantiao (GB 30), Zusanli (ST 36), Yanglingquan (GB 34), and Kunlun (BL 60).

**Needling technique:** After routine disinfection of the acupoints, standard needling is performed, ensuring “Deqi” sensation is achieved at all points. Electrical stimulation is then applied using pairs of needles at the following combinations: lumbar X-B2 and the affected side’s BL 25 – GB 30, ST 36 – GB 34. Continuous wave stimulation at a frequency of 2 Hz and an electrical current intensity ranging from 0.1 to 1.0 mA, adjusted to the patient’s tolerance, with needles retained for 30 min. Infrared lighting is also applied to the lumbar area during treatment.

**Treatment regimen:** Same as the experimental group.

The location of acupoints followed the World Health Organization’s Standard Acupuncture Locations (27).

### 2.7 Western medicine treatment for all included patients

All patients included in this study received basic conventional Western medicine treatment: starting from the second day after surgery, they were administered anti-inflammatory and analgesic treatment (celecoxib capsules 200 mg, 1 capsule/time, 2 times/day) combined with neurotrophic treatment (mecobalamin tablets 0.5 mg, 1 tablet/time, 3 times/day) for 28 days (Note: Patients were required to continue treatment at the outpatient clinic after discharge. If their numbness symptoms disappeared, the basic Western medicine treatment was discontinued, which was recorded in the experimental protocol, and follow-up was continued according to the experimental protocol).

Drug Sources:

1. Mecobalamin Tablets: Produced by Jiangxi Qingfeng Pharmaceutical Co., Ltd., Approval Number: Guoyao Zhunzi H20051440.
2. Celecoxib Capsules: Produced by Pfizer Pharmaceutical Co., Ltd., Approval Number: Guoyao Zhunzi J20120063.

### 2.8 Outcomes measurement

#### 2.8.1 Primary outcome

**Figure 3:**
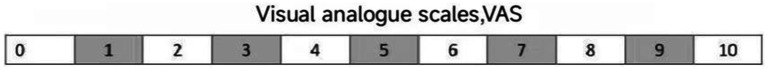
Visual analog scale for numbness.

Numbness VAS Score: Patients are asked to mark their level of numbness on a 10 cm horizontal line. A higher score indicates a more severe degree of numbness. A score of 0 indicates no numbness; 1–3 indicates mild numbness that does not affect daily life; 4–6 indicates moderate numbness with a decreased quality of life but still self-care ability; 7–9 represents severe numbness with loss of self-care ability; and a score of 10 indicates intense, unbearable numbness (see Figure 3).

#### 2.8.2 Secondary outcome

Nylon Filament Test (10 g Monofilament Test): A 10 g monofilament is used to stroke the skin area where the patient feels numb. The numb skin area is stroked 10 times, and the number of strokes perceived is observed and recorded. Perceiving 7–10 strokes is considered normal, 1–6 strokes is considered reduced sensation, and 0 strokes is considered absent sensation. The researcher records these as 3 points, 2 points, and 1 point, respectively. The study indicates that tactile static testing using a 10 g monofilament demonstrates the highest sensitivity for detecting superficial sensory disturbances. It can be recommended as the most sensitive single testing tool (28).

Pinprick Test (40 g Pressure Pinprick Test): A blunt-tipped pressure needle is used to apply a constant force to the patient’s numb area. The presence or absence of pain is observed and recorded, with pain scored as 1 point and no pain scored as 0 points. The experiment differentiates neuropathic pain from sensory loss by assessing Aδ fiber function, with sensitivity and specificity ranging from 68 to 85% and 79 to 92%, respectively (29). The test demonstrates good reproducibility (ICC = 0.89) in the assessment of postoperative neuropathy (30).

Other secondary indicators: JOA (Japanese Orthopedic Association) Score, a study mentioned that the JOA scoring system was used to evaluate 89 patients who underwent lumbar decompression surgery, and it was found that 30.3% of patients still had mild lower extremity pain/numbness and 14.6% of patients still had gait disturbances 2 years after the surgery. This indicates that the JOA scoring system can effectively identify residual symptoms after surgery (31). Traditional Chinese Medicine Syndrome Score, the TCM Syndrome Score does not focus solely on individual symptoms, but rather considers multiple symptoms and signs comprehensively, providing a more holistic assessment of the patient’s condition, it has high effectiveness in evaluating treatment outcomes (32). Additionally, Lower Limb Electromyography (H-reflex difference, F-wave conduction velocity, and latency), VAS Score, SF-36 (Short Form 36 Health Survey Questionnaire). The evaluation time points are detailed in Table 1.

**Table 1:** Timeline for the collection of evaluation indicators.

Observation indicators	Pretreatment	Treatment stage		Follow-up phase	
	Baseline (in geodetic survey)	1 week	4 weeks	12 weeks after treatment	24 weeks after treatment
General information	√				
Numbness VAS score	√	√	√	√	√
Nylon rope test	√	√	√		
Needle punching test	√	√	√		
JOA score	√	√	√	√	√
Lower Limb EMG	√	√	√		
Chinese medicine evidence score	√	√	√	√	√
SF-36 (Quality of Life Scale)	√	√	√	√	√
VAS score	√	√	√	√	√
Safety observations		√	√	√	√

### 2.9 Safety assessment

During the study, observations and records will be made of any potential adverse reactions, such as bleeding caused by acupuncture, hematoma, needle sickness, pain at the acupuncture site, infection, as well as skin rupture and infection after moxibustion. The primary records will include the incidence rate of adverse events and the grading of safety evaluations. Adverse events will be graded according to the Common Terminology Criteria for Adverse Events (CTCAE) Version 5.0. Mild (Grade 1): asymptomatic or mild symptoms, requiring no intervention; Moderate (Grade 2): symptoms are evident and require non-invasive treatment (such as local hemostasis, disinfection, etc.); Severe (Grade 3 and above): symptoms are severe, requiring hospitalization or leading to discontinuation of treatment. The evaluation time points are detailed in Table 1.

### 2.10 Data collection and management

After participants sign the informed consent form, researchers will collect personal information such as general demographic information, clinical history, as well as baseline data including the Numbness VAS Score, 10 g Monofilament Test, 40 g Pressure Pinprick Test, JOA Score, Lower Limb Electromyography (H-reflex difference, F-wave conduction velocity, and latency), VAS Score, Traditional Chinese Medicine Syndrome Score, SF-36 (Short Form 36 Health Survey Questionnaire), etc. Outcome measures will be assessed at specified time points and recorded in the CRF. After the trial, two researchers who are not involved in data distribution or outcome assessment will independently extract data from the CRF into an Excel table. A statistician will then perform statistical analysis on this data after careful review and confirmation. To protect participant privacy, personal names will be replaced with a combination of numbers and initials. Researchers shall keep the data confidential for 5 years after the trial termination. To reduce participant dropout rates, researchers will call participants 2 days before treatment to inquire about their condition and encourage them to receive treatment as scheduled.

### 2.11 Quality control

A coordination management team, led by the project leader, shall be established, with dedicated trial auditors and inspectors responsible for trial management and quality control. To minimize bias in human operations, all researchers involved will undergo training. The entire research process will be supervised and inspected to ensure that all operations are carried out by standard operating procedures and that the recording and reporting of research data, treatment procedures, outcome assessments, management of investigational drugs, adverse events, etc., are filled in truthfully, accurately, and completely. Prompt measures will be taken to address any identified quality issues. The scientific research office of the project undertaking unit shall bear the responsibility for project management.

### 2.12 Statistical analysis

Sample size calculation was performed using PASS software (version 15.0, NCSS LLC). Based on preliminary data, the estimated mean (standard deviation) of the VAS numbness score after intervention for the treatment group (UA + WA) is 1.6 ± 1.0 cm, and for the control group (electroacupuncture) it is 2.41 ± 1.68 cm. To detect a between-group difference of 0.81 cm with 90% power (*β* = 0.10) and a two-sided *α* of 0.05, at least 41 participants are required in each group. Considering a 15% dropout rate, the final sample size is set at 48 participants per group (96 participants in total). This calculation complies with the CONSORT guidelines for randomized controlled trials.

All analyses will follow the intention-to-treat (ITT) principle, including all randomized participants. Additionally, for sensitivity analysis, a per-protocol set (PPS) analysis will be conducted, excluding participants with major protocol deviations (e.g., fewer than 8 acupuncture sessions, and use of prohibited medications). The primary outcome, the change in VAS numbness score over time points (baseline, 1 week, 4 weeks, 12 weeks, 24 weeks), will be assessed using a linear mixed-effects model (LMM). The model will include group, time, group-by-time interaction, and baseline VAS score as fixed effects. Random intercepts and slopes will account for within-individual variability. Missing data will be handled using multiple imputation (MI) with fully conditional specification (FCS) in SAS 9.4 (SAS Institute), generating 10 imputed datasets. Estimates will be combined according to Rubin’s rules. Sensitivity analysis will compare MI with the last observation carried forward (LOCF). For secondary outcomes with continuous variables (e.g., JOA, SF-36), the same LMM structure as the primary outcome will be used. For ordinal/categorical variables (e.g., TCM syndrome score), proportional odds logistic regression or generalized estimating equations (GEE) will be applied to longitudinal ordinal data. For binary outcomes (e.g., adverse events), comparisons between groups will be made using the chi-square test or Fisher’s exact test (for small sample sizes). Risk ratios (RR) and 95% confidence intervals (CI) will be reported.

The success of assessor blinding will be evaluated using James’ blinding index. A value close to 0.5 indicates effective blinding (0 = completely unblinded; 1 = completely blinded). If there are baseline differences (e.g., age, baseline VAS score), adjustments will be made using analysis of covariance (ANCOVA). Pre-specified subgroups (e.g., age < 50 vs. ≥ 50 years, baseline VAS score severity) will be explored using interaction terms in regression models. *p*-values will be adjusted using the false discovery rate (FDR) correction for multiple comparisons for the primary outcome.

Adverse events (AE) will be graded according to CTCAE v5.0. The incidence rates between groups will be compared using the chi-square test (or Fisher’s exact test for rare events). Descriptive analysis will be conducted for serious adverse events (≥ grade 3). Analyses will be performed using SAS 9.4 (SAS Institute) and SPSS 26.0 (IBM Corp). Continuous outcomes will be reported as mean differences (MD) with 95% CI and effect sizes (Cohen’s d). Categorical outcomes will be reported as risk ratios (RR) with 95% CI. Exact *p*-values will be provided for all tests, with a significance level set at *p* < 0.05 for the primary outcome.

### 2.13 Dissemination

The results of the study will be presented at scientific conferences and in peer-reviewed publications. Participants included in the study will also have the opportunity to obtain the study results by telephone or e-mail.

### 2.14 Trial status

Currently, the protocol is version 1.0, registered on 31 August 2024. At the time of protocol submission, potential participants of the study have been actively enrolled.

## 3 Discussion

This experiment is a single-center, prospective, randomized, patient-assessor-blinded, controlled trial that aims to evaluate the effectiveness and safety of UA combined with WA in treating residual numbness after LDH surgery. From a modern medical perspective, it falls under the category of peripheral nerve disorders.

Drawing from the perspective of traditional Chinese medicine (TCM) pathogenesis and building on our prior clinical practice, we have, for the first time, applied UA combined with WA to treat postoperative numbness. UA is an emerging therapy established by Professor Qi Yong. Its theoretical basis is completely rooted in traditional Chinese culture. UA is different from traditional acupuncture, which is a therapeutic method to treat diseases by acupuncture at the umbilical point in a certain way based on the theory of bioholography (33). Compared with other acupuncture methods, umbilical acupuncture (UA) regulates the body’s qi and blood through the holographic reflex zones of the umbilicus, characterized by fewer acupoints and milder stimulation. Electro-acupuncture primarily exerts its effects through local nerve regulation, with relatively weak overall regulatory effects on systemic functions. Warm needle acupuncture that combines moxibustion and acupuncture has become increasingly popular due to its significant clinical efficacy and safety (34). This approach treats both the symptoms and the root causes, with a particular emphasis on addressing the underlying causes. Some studies have shown that WNA has better effects in relieving pain and promoting blood circulation compared with simple acupuncture or moxibustion (35). The therapeutic mechanism of WA is similar to the process of thermotherapy, in which a high-temperature area is formed locally in a tissue to promote metabolism, enlarge blood vessels, and reduce the excitability of peripheral nerves (34). The stimulus from needles in acupuncture involves a change of temperature and heat transfer through tissue. Both ambient and body temperatures influence the effect of acupuncture, this stimulus may result in the regulation of homeostasis (36). Comparing the physical efficiency of acupuncture needles regarding heat transfer with the daily energy consumption of a human body reveals an interesting and new aspect: Applying the normal energy consumption rate of a human body to CO3 domain volume results in 0.00003262 W, showing that the amount of energy conveyed by acupuncture needles is much higher than the body’s energy consumption and suggesting a strong stimulating effect in the regulation of homeostasis (36). UA and WA are widely used in clinical practice and have been proven safe (33).

According to domestic literature reports, electroacupuncture has a definite therapeutic effect in treating residual pain and numbness after percutaneous transforaminal endoscopic discectomy (PTED) for LDH. The overall effective rates for pain and numbness relief are 95.45 and 92.86%. The overall efficacy of combined electroacupuncture treatment is superior to that of Western medicine treatment alone (37). Some other articles have reported that low-frequency EA was applied directly to the spinal nerve root at right L4, L5, and, S1, immediately after treatment, right or left back and lower extremity pain and numbness were markedly reduced. The walking distance of patients was also greatly extended. The mechanisms underlying this effect may be attributed to inhibitory control at the spinal level, inhibition of potential activity by hyperpolarization of nerve endings, or changes in nerve blood flow (38, 39).

Therefore, we have established an electroacupuncture control group. We will compare this treatment with electroacupuncture therapy to explore a potentially more effective acupuncture treatment regimen that promotes early rehabilitation for patients with numbness after LDH surgery. We hope to find a better acupuncture treatment method to guide clinical practice and improve treatment outcomes. In addition, this study may provide more scientific evidence for non-pharmacological treatments and facilitate future related research.

This study has certain limitations. Its single-center design may not fully represent the clinical practice situation across multiple centers. The VAS (Visual Analog Scale) score is a subjective outcome measure that is susceptible to individual differences and psychological factors among patients. The follow-up period of this study is 6 months, which may not be sufficient to fully assess the long-term effects of the treatment. Insufficient sample size may affect the statistical power of the results, and there may be issues with poor patient compliance and high dropout rates in the study.
